# The Synthesis and Characterisation of a Molecular Sea‐Serpent: Studies of a {Cr_24_Cu_7_} Chain

**DOI:** 10.1002/anie.202015731

**Published:** 2021-03-17

**Authors:** Rajeh Alotaibi, Jonathan M. Fowler, Selena J. Lockyer, Grigore A. Timco, David Collison, Jürgen Schnack, Richard E. P. Winpenny

**Affiliations:** ^1^ Department of Chemistry The University of Manchester Oxford Road Manchester M13 9PL UK; ^2^ Faculty of Physics Bielefeld University P.O. box 100131 33501 Bielefeld Germany

**Keywords:** chromium, magnetic studies, spin chains, supramolecular

## Abstract

A finite chain of thirty‐one paramagnetic centers is reported, synthesized by reaction of hydrated chromium fluoride, copper carbonate and pivalic acid in the presence of 1,4,7,10‐tetrazacyclododecane (cyclen). Magnetic studies show predominantly anti‐ferromagnetic exchange leading to a high density of low‐lying spin states and large saturation field.

## Graphical abstract

The longest finite chain of metals is reported and the magnetic properties studied and modelled.

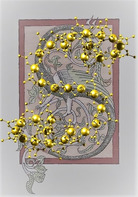

The designed approach to the synthesis of polymetallic complexes often uses rigid ligands with donor atoms organised to bind to metal sites with specific geometries.[^1^, ^2^, ^3^, ^4^, ^5^, ^6^] The design uses our knowledge of the preferred coordination geometries of metals and that a ligand is bi‐ or tri‐dentate, rigid or flexible. Templates can also be used to direct complexes towards specific metal nuclearities.[^7^, ^8^]

Another route recognises that there are always chemical factors that are outside our complete control, and allowing such factors to play a part in reaching new structures. In this paper we take a well‐studied system,^[9]^ and include two reactants that lead to an unusual chain compound.

We have been studying the formation of cyclic and acyclic complexes from hydrated chromium fluoride reacted with pivalic acid in the presence of an amine and a divalent metal ion that favours a regular octahedral coordination environment, typically nickel(II).^[9]^ Where the amine is a secondary amine with linear side‐chains, for example, di‐*n*‐propylamine, we form in very high yield a compound [^n^Pr_2_NH_2_][Cr_7_NiF_8_(O_2_C^t^Bu)_16_].^[10]^ The formation of this eight‐metal ring can be easily rationalised. For example: the bond angles at the six‐coordinate metal sites lead to octahedral coordination geometries without strain; the carboxylates can adopt their preferred bridging mode; the fluorides bridge two metals; the protonated ammonium cation H‐bonds to the fluorides and acts as a template for the ring. If we make the side‐chains on the amine more sterically demanding, or use a protonated N‐heterocycle, nine‐ or ten‐metal versions of this structure result.[^11^, ^12^, ^13^] The building block is always the {M_2_F(O_2_C^t^Bu)_2_} fragment, which can oligomerise readily. The eight‐metal ring is the smallest closed structure, and hence favoured by entropy. Entropic control matches the reaction temperature of 140° C in molten pivalic acid.

Inclusion of copper(II) in place of nickel(II) leads to a series of larger rings {Cr_*x*_Cu_2_} (where *x=*10, 11 or 12}, which can be rationalised as due to the propensity of Cu^II^ to show five‐coordinate geometry rather than six‐coordinate.^[14]^ If we replace the simple amine by cyclen we find it coordinates to Ni^II^ producing an S‐shaped molecule, [{Ni(cyclen)}_2_Cr_12_NiF_20_(O_2_C^t^Bu)_22_] which we have described as a molecular seahorse, **1**.^[13]^ We can rationalise this as coordination of the tetradentate ligand to Ni^II^ leads to it acting as a terminal group and preventing ring closure. Another factor is the crystal field stabilisation energy for nickel(II); once bound to the cyclen it tends to stay bound and will adopt a six‐coordinate geometry.

Combining both Cu^II^ as the divalent ion and cyclen as the template introduces a further degree of uncertainty in our design and here it leads to a remarkable result. The reaction of chromium(III) fluoride hydrate, cyclen, basic copper carbonate, and pivalic acid at 140–160 °C for 5 h produced [Cu(H_2_O)(cyclen)]_2_[Cr_24_Cu_5_{Cu(cyclen)}_2_F_40_(O_2_C^t^Bu)_50_], **2**. It was obtained as an isolated product with low but reproducible yield, and can be separated from the neutral byproduct [CrF(O_2_C^t^Bu)_2_]_8_ by extraction or crystallization. Crystals can be grown from a mixture of Et_2_O/MeCN solvent. The structure of **2** (Figure 1) is related to the S‐shaped {Cr_12_Ni_3_} complex^[13]^ but is far longer. We are unaware of any finite 1D‐chain containing more metal centres. An {Fe_18_} chain has been reported by Christou and co‐workers.^[14]^

**Figure 1:**
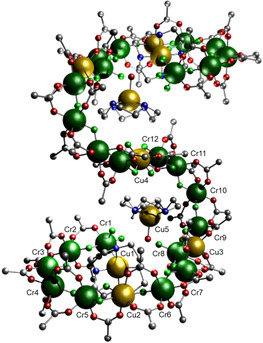
The structure of **2** in the crystal. Color scheme: Cr, green; Cu, yellow; F, light green; N, blue; O, red; C, black. H‐atoms and methyl groups of pivalate omitted for clarity.^[20]^

The asymmetric unit comprises one half of the oligomer, with the central copper (Cu4) residing on an inversion centre. There are seven Cu^II^ sites within the 31‐metal chain. Cu5, and its symmetry equivalent are bound to cyclen and a water and is not attached to the chain. The other Cu sites are linked by a variety of Cr‐F fragments within the chain. Beginning at Cu1, there is a {Cr_5_} chain (Cr1…Cr5) that links to Cu2. There is then a {Cr_3_} (Cr6…Cr8) that links to Cu3, and finally a {Cr_4_} chain (Cr9…Cr12) that links to the central Cu4 site. We have only previously seen more than one Cr‐chain in the same molecule in a {Cr_11_Cu_2_} ring.^[15]^

Each Cr⋅⋅⋅Cr contact in the three distinct chains is bridged by a fluoride and two pivalates, the same motif we have seen in previous rings and chains.[^9^, ^10^, ^11^, ^12^, ^13^, ^15^] The linking of the Cu sites to the Cr chains varies. Cu1 that terminates the chain (see Figure 2) is five‐coordinate, adopting a square‐pyramidal geometry, bound to 4 N‐atoms from cyclen in a square with a fluoride on the axial site bridging to Cr1. The Cu−F bond length of 2.094(7) Å is longest establishing this as the electronic *z*‐axis for the Cu^II^ site. Bond length ranges are given in Table 1.

**Figure 2:**
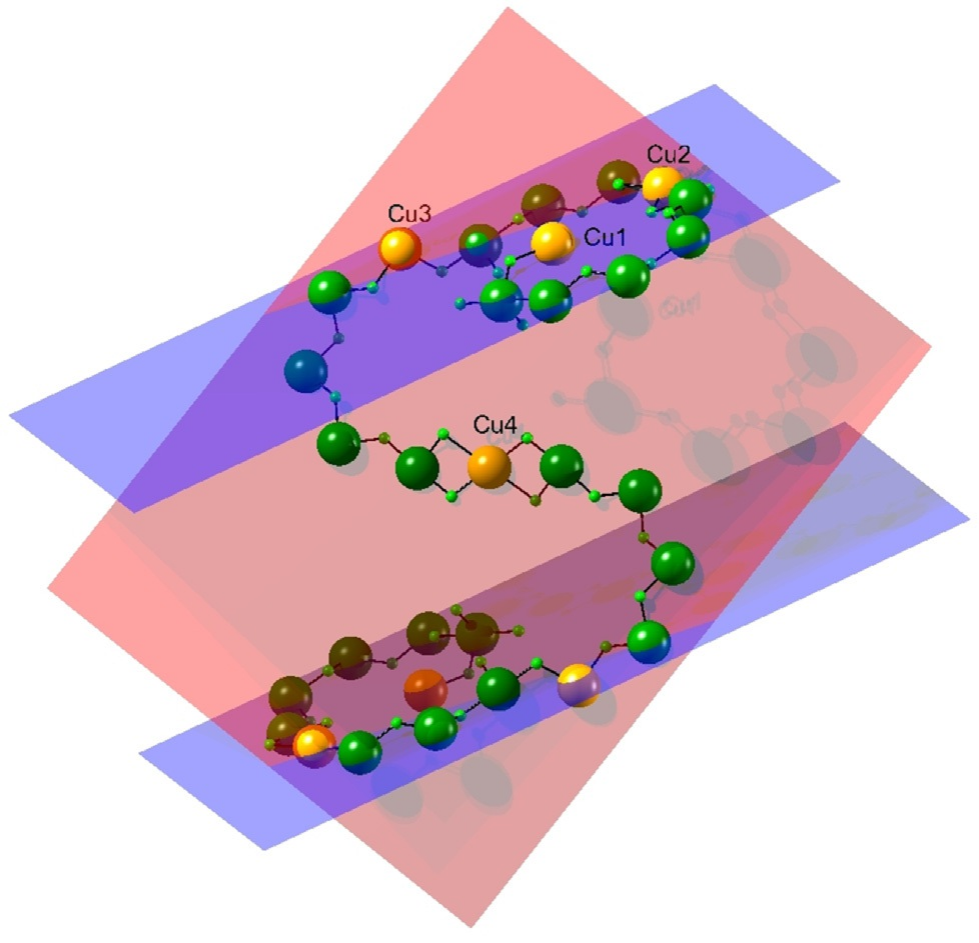
The core of **2** illustrating the central {Cr_8_Cu_3_} S‐shape and the external {Cr_8_Cu} units. The main plane of the central S‐shape shown as peach, the mean planes of the external {Cr_8_Cu} units in lilac. Only Cr, Cu and F included with color scheme as Figure 1.

**Table 1:** Bond length ranges (Å) in **2**.

Metal site	Coordination environment	F‐bond length range^[a]^/Å	O‐bond length range^[b]^/Å
Cu1 Cu5	1F 4N 1O 4N	2.094(6) 2.139(8)	1.993(11)–2.009(11) 2.005(10)‐2.028(9)
Cu2	3F 3O	1.954(10)–2.252(14)	1.884(15)–1.994(13)
Cu3	2F 3O	1.953(6)–2.211(7)	1.894(13)–1.916(10)
Cu4^[c]^	4F 2O	2.318(6)	1.943(8)–1.947(7)
Cr1	4F 2O	1.881(7)–1.943(7)	1.935(11)–1.991(9)
Cr5^[d]^ Cr8 Cr12	3F 3O	1.826(10)–2.040(16) 1.878(6)–1.898(7) 1.875(7)–1.911(6)	1.916(17)–2.02(4) 1.892(11)–1.898(7) 1.938(11)–1.984(10)
Other Cr	2F 4O	1.863(9)–1.941(8)	1.850(15)–2.04(2)^[d]^

[a] Except Cu5 where the Cu−O bond length given. [b] Except Cu1 and Cu5 where Cu−N bond length given. [c] On an inversion centre. [d] Disorder in one O‐donor site.

Cu2 is bridged by two fluorides and a pivalate to Cr5 and by a single fluoride and two pivalates to Cr6. Two of the fluorides are on the elongated Jahn–Teller (JT) axis. Cu3 is bridged to Cr8 by a single fluoride and single pivalate, and to Cr9 by a fluoride and two carboxylates; this latter fluoride is on the JT axis. Cu4 is on the inversion centre and is bridged to Cr12 by two fluorides and a single pivalate. The JT‐axis for Cu4 is to two F‐atoms.

Cu5 is part of a [CuF(cyclen)]^+^ cation, which forms N‐H⋅⋅⋅F hydrogen bonds to the chain with N⋅⋅⋅F separations of 2.8–2.9 Å.

The five unique copper sites have four distinct geometries. Cu1 and Cu5 are square pyramidal, the former bound to 4N and 1F and the latter to O4 and 1O. Cu3 is also square pyramidal but bound to two F and three O donors, as noted above the fluoride is on the apex of the square pyramid. Cu2 is six‐coordinate with a *mer*‐arrangement of three O and three F donors, while Cu4 is six coordinate with a *trans*‐arrangement of four F and two O donors. This capability of Cu^II^ to adopt five different coordination environments in one complex is a major reason for the formation of **2**. All the Cr^III^ sites are six‐coordinate, with Cr2, Cr3, Cr3, Cr4, Cr6, Cr7, Cr9, Cr10 and Cr11 having a *cis*‐arrangement of two F and four O donors. Cr5, Cr8 and Cr12 have a *mer*‐arrangement of three O and three F donors. Cr1 is unique in having four F and two O‐donors with a *cis*‐geometry.

No significant intermolecular interactions were observed in the solid state, which is similar to related rings.^[9]^ This is a consequence of the chain possessing fifty bridging pivalate groups, which effectively block any interactions between the neighbouring chains.

There is considerable resemblance between compounds **1** and **2**. In **1** all nickel sites are six‐coordinate and it is the coordinative flexibility of Cu^II^ that allows formation of the longer chain. Examining the core of **2** we can see an almost planar metal S‐chain {Cr_8_Cu_3_} running from Cu3 to its symmetry equivalent via two {Cr_4_} chains and Cu4 (the plane shown in peach in Figure 2). This is very similar to **1**, but in **1** there are {Cr_6_} chains giving {Cr_12_Ni_3_}. The central Ni site in **1** has the same coordination environment as Cu4 in **2**. In **2** beyond Cu3 there is a further planar {Cr_8_Cu} unit (the plane shown in lilac in Figure 2); the mean plane of this unit is at 118° to the plane of the central {Cr_8_Cu_3_} S‐shape.

Magnetic studies of **2** show predominantly anti‐ferromagnetic exchange is present. Susceptibility (*χ_m_*) was measured from 300 to 2 K in a 5000 Oe field and the magnetisation *(M*) was measured from 0 to 7 T at various temperatures (Figure 3). The product *χ_m_T* shows a steady decline with falling temperature, while *M* increases steadily with field at all temperatures in the range 2 to 4 K, and has not saturated even at the lowest *T* and highest *H*. As expected, this is consistent with multiple low‐lying paramagnetic states as well as sizable antiferromagnetic exchange for this long chain of unpaired spins.

**Figure 3:**
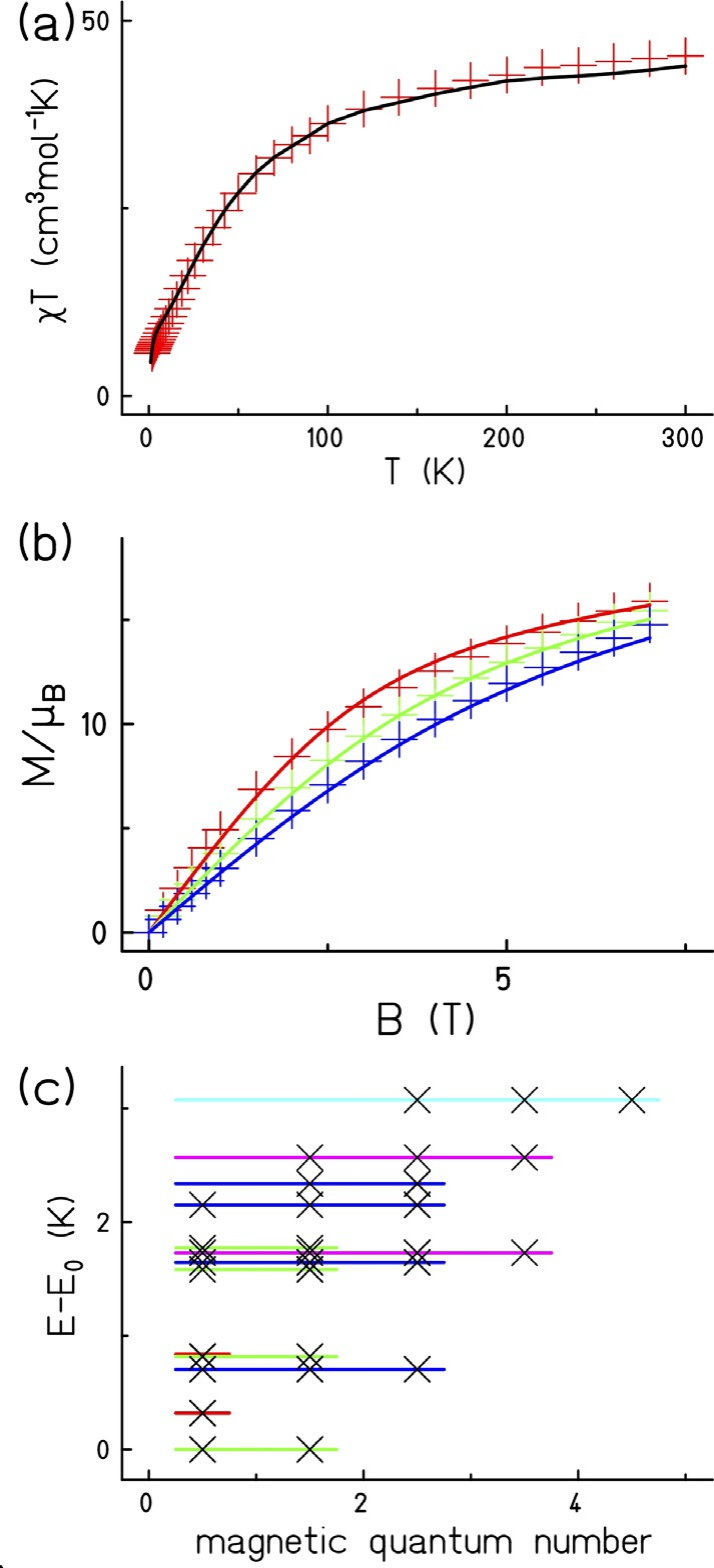
Magnetic studies of **2**. (a) Measured (red symbols) and fitted (black solid curve) *χ_m_T* vs. *T*. (b) measured and fitted *M* vs. *B* at 2 (red symbols and line), 3 (green) and 4 (blue) K. (c) Low‐lying DMRG energy eigenvalues: for each magnetic quantum number (>0) the lowest five levels were evaluated. These belong to multiplets marked by colored lines: *S=*1/2 (red), *S=*3/2 (green), *S=*5/2 (blue), *S=*7/2 (magenta) and *S=*9/2 (light blue). Fits used parameters given in Table 2 and text.

To fit the magnetic data of such a large molecule is not trivial, however because there is precedent for many of the super‐exchange pathways then fitting is possible. We first made the assumption that Cu5 and its symmetry equivalent act as independent *s*=1/2 centers and are not coupled to the ring. The number of super‐exchange paths was kept to five, as listed in Table 2, giving the Hamiltonian 1 for the 31 coupled magnetic centres, that is, without Cu5 and Cu5′

(1) $$\hat{H}=\;\sum_{i=1}^{30}J_{i,i+1}\;{\hat{\vec{s}}}_{i}\cdot {\hat{\vec{s}}}_{i+1}+g\mu_{B}B\sum_{i=1}^{31}{\hat{s}}_{i}^{z}$$

**Table 2:** Exchange interactions used in Hamiltonian (1) to fit *χ_m_T*(*T*) and *M*(*H*).

Label	Bridge	Contact	Value from Precedent^[16]^/ K
*J*_1_	One F on *z*‐axis No carboxylates OR One F on *z*‐axis Two carboxylates	Cu1‐Cr1 Cu2‐Cr6; Cu3‐Cr9	−21
*J*_2_	One F Two carboxylates	All Cr‐Cr	16
*J*_3_	Two F in *xz*‐ plane One carboxylate	Cr5‐Cu2	Variable
*J*_4_	One F in *xy*‐plane One carboxylate	Cr8‐Cu3	55
*J*_5_	Two F in *xz*‐plane One carboxylate	Cr12‐Cu4	Variable >0

The Sea‐Serpent belongs to the largest spin systems of finite size; the Hilbert space dimension is 36,028,797,018,963,968 (without Cu5 and Cu5′). Exact diagonalization works up to 10^5^, finite‐temperature Lanczos up to 10^10^. Fortunately, the structure is one‐dimensional and not frustrated. The first property allows us to determine low‐lying energy eigenvalues by means of density‐matrix renormalization group (DMRG) methods, and the second enables quantum Monte Carlo (QMC) in order to evaluate thermal properties such as the magnetization as a function of temperature and applied field. For both types of calculations we employed the free ALPS library.[^17^, ^18^] An average g value of 2.0 was assumed for the calculations.

There are many assumptions here, perhaps chiefly that the exchange of Cu1‐Cr1 is equivalent to that of Cu2‐Cr6 or Cu3‐Cu9; this assumption is based on the exchange being dominated by the F‐bridge on the *z*‐axis on the Cu site, regardless of the number of carboxylate bridges. In Table 2 three of the five interactions were fixed, based on a previous study of a {Cr_12_Cu_2_} ring, where the exchange interactions were determined by three techniques: magnetometry, inelastic neutron scattering and tunnel diode oscillator measurements.^[16]^ The final two parameters, *J_3_* and *J_5_* were allowed to vary with *g* fixed at 2.0. The best fit was then achieved with *J*_3_=3 K and *J*_5_=10 K (Figure 3). Given the limited data available introduction of still further parameters (e.g. making the Cu1‐Cr1 exchange a separate variable *J*_6_) is not justified.

These parameters produce a spin state structure with multiple low‐lying paramagnetic states. DMRG calculations[^17^, ^18^] give a ground state with total spin *S=*3/2. Of the many levels within 3 K of the ground state we targeted five for each magnetic quantum number (symbols in Figure 3 c). They are grouped into spin multiplets when energetically degenerate and marked according to assigned total spin. The true spectrum contains many more levels above those targeted by DMRG; it is simply increasingly complicated to obtain them with reliable accuracy.

The EPR spectra of **2** are broad with two interesting features (Figure S1). Firstly, the spectrum in frozen solution (1:1 CH_2_Cl_2_:toluene) and powder at 5 K are very similar. This suggests the structure of the chain and its supramolecular interaction with the cation containing Cu5 is maintained in solution. Secondly, there is copper hyperfine structure on the low field feature in the spectra. We believe this is due to the resonances from the isolated Cu5 complex. This lower field feature is centered at *g=*2.13 while the higher field feature is at *g=*1.960, 1.990, 1.990. The spectrum was simulated^[19]^ as the sum of a {Cr_3_} chain with *g=*1.960 and *J=*16 K and two Cu^II^ sites with *g* values of 2.075, 2.055, 2.145 and 2.065, 2.045, 2.135.

The synthesis and structure of **2** demonstrates the richness and complexity of the chemistry that is found when chromium fluoride, pivalic acid and a copper(II) salt are present.^[15]^ The yield of **2** is low, but reproducible. There must be other oligomers formed that we have not crystallized to this point. It also suggests that inclusion of other N‐donor macrocycles may lead to still further unusual structures.

## Conflict of interest

The authors declare no conflict of interest.

## Supporting information

As a service to our authors and readers, this journal provides supporting information supplied by the authors. Such materials are peer reviewed and may be re‐organized for online delivery, but are not copy‐edited or typeset. Technical support issues arising from supporting information (other than missing files) should be addressed to the authors.

Supplementary
