# Supplementary material for: The Synthesis and Characterisation of a Molecular Sea‐Serpent: Studies of a {Cr24Cu7} Chain
Source: Angew Chem Int Ed Engl. 2021 Mar 17;60(17):9489–92. doi: 10.1002/anie.202015731 (PMC8251705; doi:10.1002/anie.202015731)
Supplement: Supplementary file 1 — Supplementary [file ANIE-60-9489-s001.pdf]

Angewandte Chemie
Eine Zeitschrift der Gesellschaft Deutscher Chemiker

Supporting Information

The Synthesis and Characterisation of a Molecular Sea-Serpent: Studies of a $\{Cr_{24}Cu_7\}$ Chain

Rajeh Alotaibi, Jonathan M. Fowler, Selena J. Lockyer, Grigore A. Timco, David Collison, Jürgen Schnack, and Richard E. P. Winpenny**

anie_202015731_sm_miscellaneous_information.pdf

Supplementary Material

Synthesis

$[Cu(H_2O)(cyclen)]_2[Cr_{24}Cu_5\{Cu(cyclen)\}_2F_{40}(O_2C^tBu)_{50}]$ (2): Basic copper carbonate (1.13 g, 5.1 mmol), deionized water (1 g, 55 mmol), and $^tBuCO_2H$ (15 g, 147 mmol) were heated to 140 ºC with stirring for 1 h. Cyclen (0.7 g, 4.0 mmol) was added to the mixture and stirred for a further 30 mins. $CrF_3.4H_2O$ (5.0 g, 28 mmol) was added to the blue solution and the reaction was stirred at 160 ºC for 5 h. The solution was cooled to room temperature, acetone (100 mL) was added and the reaction stirred overnight. The product was filtered and washed with a large quantity of acetone. The product was extracted into THF (250 mL), filtered and the filtrate solvent was removed *in vacuo*, affording the title product as a light blue solid. Yield 0.20 g (2%, calculated from the available Cr). Elemental analysis (%): calculated for $C_{282}H_{550}Cr_{24}Cu_9F_{40}N_{16}O_{102}$: Cr 14.90, Cu 6.83, C 40.43, H 6.62, N 2.68; found: Cr 14.52, Cu 6.54, C 39.86, H 6.38, N 2.65. The product was recrystallized from Et_2O/MeCN and gave X-ray quality crystals in two days.

An improved yield of 5%, calculated from the available Cr (0.50 g), was achieved by omitting the addition of deionized water.

Crystallography

Crystallographic data and refinement parameters are given in Table S1.
Data for **2** were collected using a using a dual source Rigaku FR-X rotating anode diffractometer with a HyPix 6000HE detector and microfocus optics with CuKα radiation (λ = 1.5418 Å) at 100 K. Crystals were mounted on the goniometer using Fomblin ® oil and placed under a stream of N_2. All data were and reduced using CrysAlisPro v40.[39] Absorption correction was performed using empirical methods (SCALE3 ABSPACK) based upon symmetry-equivalent reflections combined with measurements at different azimuthal angles.[39] The structure was solved using direct methods and refined against F^2 using SHELXT and SHELXL, respectively.

Crystallographic refinement details

A number of pivalates were disordered, with either the methyl groups, the tert-butyl group or in one case, the entire pivalate group being disordered over two sites. Carbon atoms were modelled with bond distance SADI and DFIX restraints where appropriate. Atomic displacement parameters of some pivalate groups were also restrained using SIMU and RIGU commands. Three MeCN solvent sites were located in the difference map, one of which was refined at 0.5 occupancy. Two were refined as isotropic, and were modelled using DFIX, RIGU and SIMU commands.

Table S1: Crystallographic data and refinement parameters for compound **2**

	**2**
Empirical formula	$C_{292}H_{549}Cr_{24}Cu_9F_{40}N_{21}O_{102}$
Formula weight	8566.35
Temperature/K	99.9(8)
Crystal system	orthorhombic
Space group	Pbca
a/Å	36.5643(11)
b/Å	20.6750(5)
c/Å	60.4525(14)
α/°	90
β/°	90
γ/°	90
Volume/Å^3	45700(2)
Z	4
ρ_{calc}g/cm^3	1.245
μ/mm^{-1}	5.616
F(000)	17844.0
Crystal size/mm^3	0.614 × 0.37 × 0.178
Radiation	Cu Kα (λ = 1.54184)
2Θ range for data collection/°	2.922 to 117.864
Index ranges	-21 ≤ h ≤ 40, -22 ≤ k ≤ 22, -67 ≤ l ≤ 65
Reflections collected	157503
Independent reflections	32746 [R_{int} = 0.1268, R_{sigma} = 0.0986]
Data/restraints/parameters	32746/997/2312
Goodness-of-fit on F^2	1.067
Final R indexes [I>=2σ (I)]	R_1 = 0.1128, wR_2 = 0.3090
Final R indexes [all data]	R_1 = 0.2166, wR_2 = 0.3807
Largest diff. peak/hole / e Å^{-3}	1.33/-0.43

Magnetic measurements

The ALPS library[4,5] was used to calculate magnetic properties by both DMRG and QMC methods. An average *g*-value of 2.00 was assumed for all calculations. For DMRG: The respective ALPS routine dmrg executed 10 sweeps ("SWEEPS=10") for each of eight increasing values – 100, ..., 800 – of the variable MAXSTATES. For QMC: The ALPS routine dirloop_sse was run for each temperature value with THERMALIZATION=10,000,000, SWEEPS=100,000,000, and EPSILON = 0.01.

The best fit is shown in the main manuscript. In Figures S1 – S5 the result of varying each parameter in turn by +/-10% are shown.

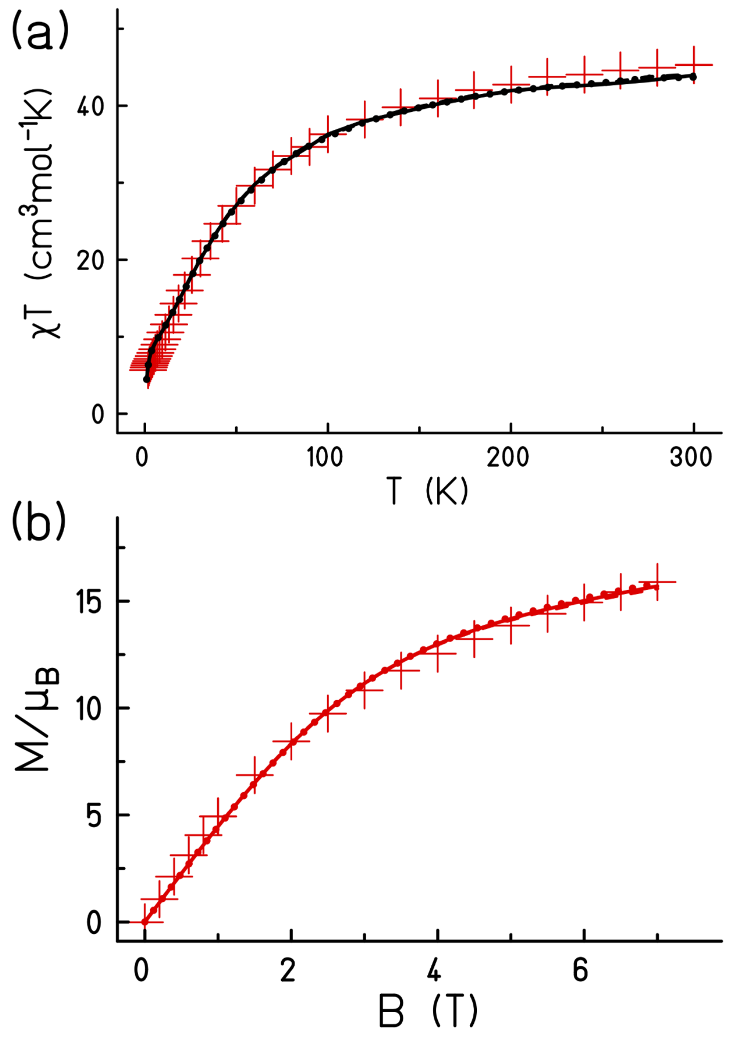

Figure S1: Variation of J_1 by +10% (dashed curves) and -10% (dotted curves) compared to fit shown in Figure 3 (solid curves). The magnetization is only shown for $T = 2$ K.

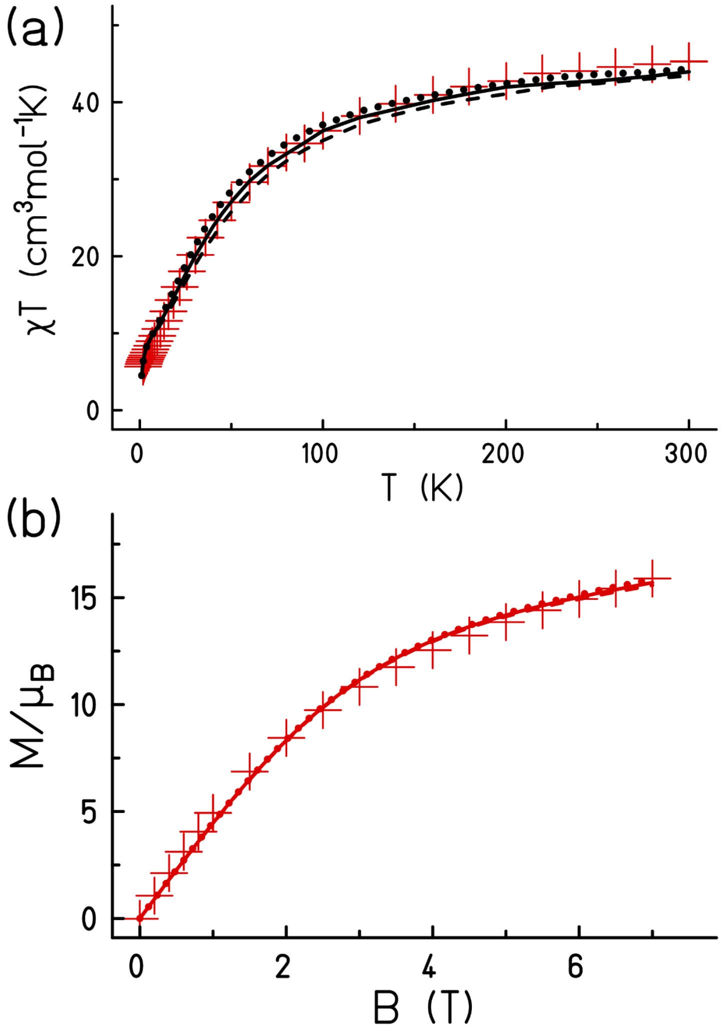

Figure S2: Variation of J_2 by +10% (dashed curves) and -10% (dotted curves) compared to fit shown in Figure 3 (solid curves). The magnetization is only shown for $T = 2$ K.

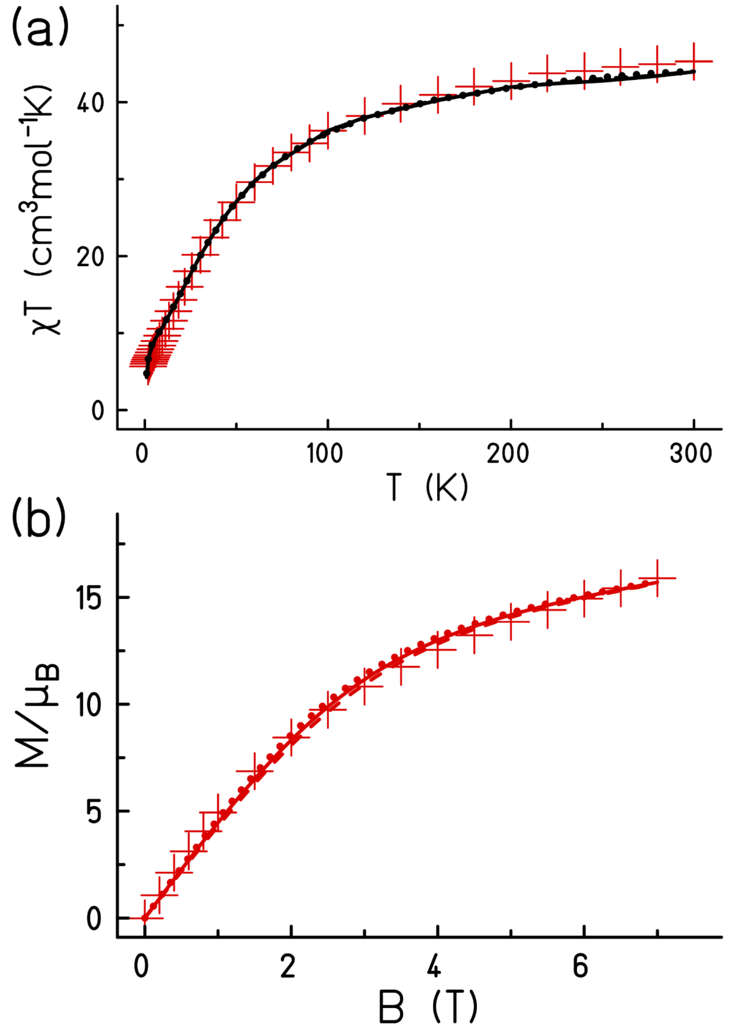

Figure S3: Variation of J_3 by +10% (dashed curves) and -10% (dotted curves) compared to fit shown in Figure 3 (solid curves). The magnetization is only shown for *T = 2* K.

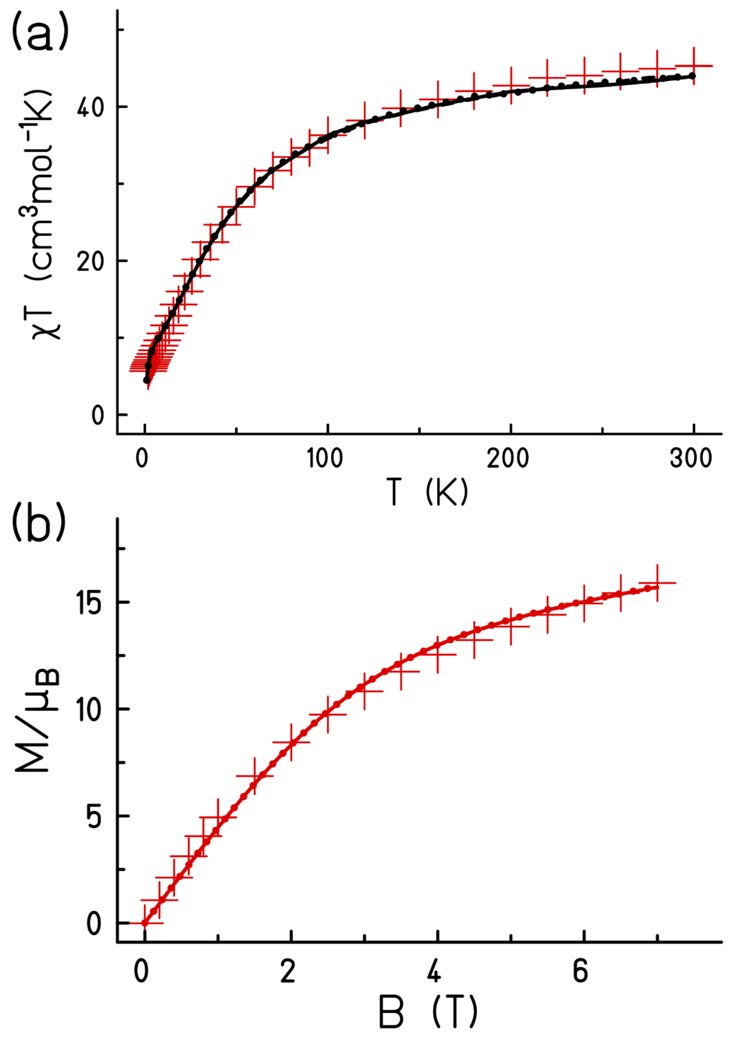

Figure S4: Variation of J_4 by +10% (dashed curves) and -10% (dotted curves) compared to fit shown in Figure 3 (solid curves). The magnetization is only shown for $T = 2$ K.

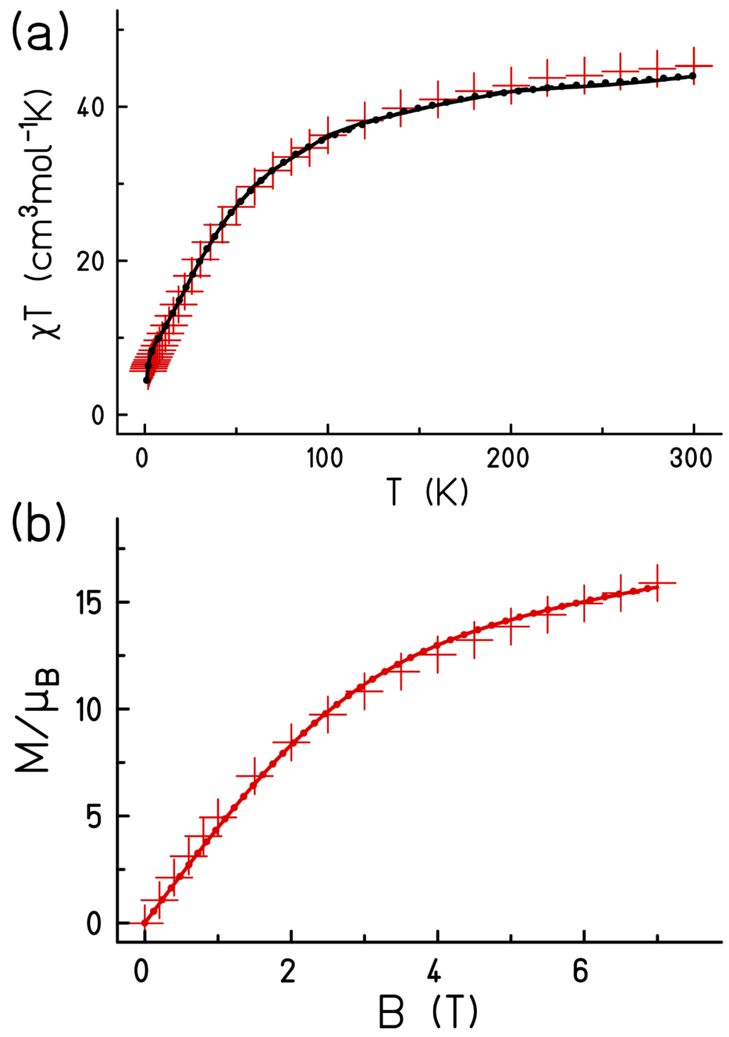

Figure S5: Variation of J_5 by +10% (dashed curves) and -10% (dotted curves) compared to fit shown in Figure 3 (solid curves). The magnetization is only shown for $T = 2$ K.

EPR spectroscopy

Continuous wave Q-band (~34 GHz) EPR spectra were recorded with a Bruker EMX580 spectrometer. Data were collected on a polycrystalline powder and a dry solution of 1:1 toluene / DCM at 5 K (unless otherwise stated) using liquid helium cooling. All continuous wave spectra were field corrected using a 'Strong Pitch' standard (g = 2.0028) and all powder samples were checked for any polycrystalline nature. Spectral simulations were performed using the EasySpin 5.2.25 simulation software [Reference A] unless stated otherwise.

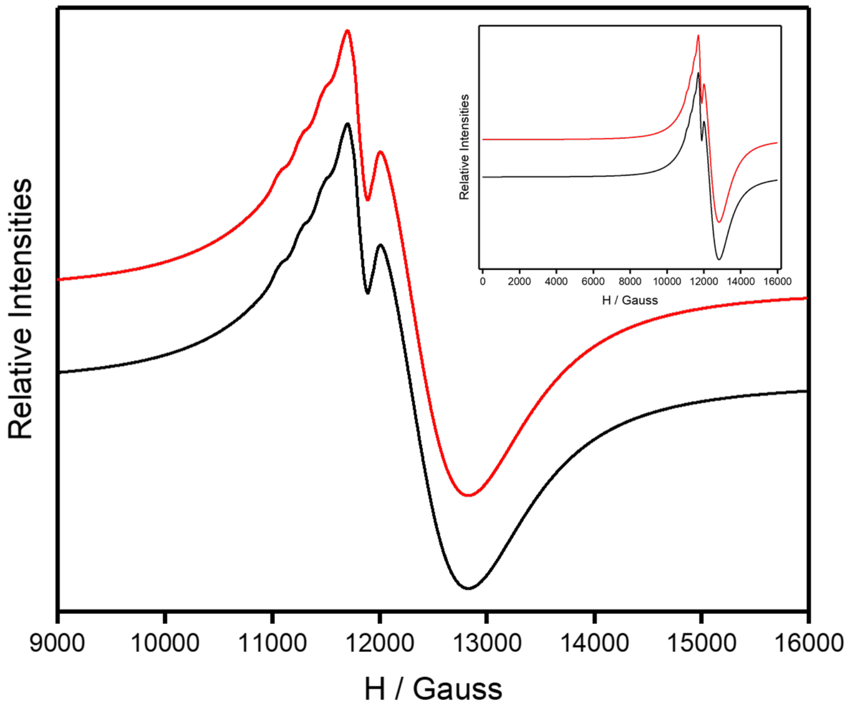

Figure S6. Continuous Wave Q-Band (ca. 34 GHz) EPR spectrum of **2** as a powder (black) at 5K and simulation (red), Insert (full field sweep, 0-16000 gauss).

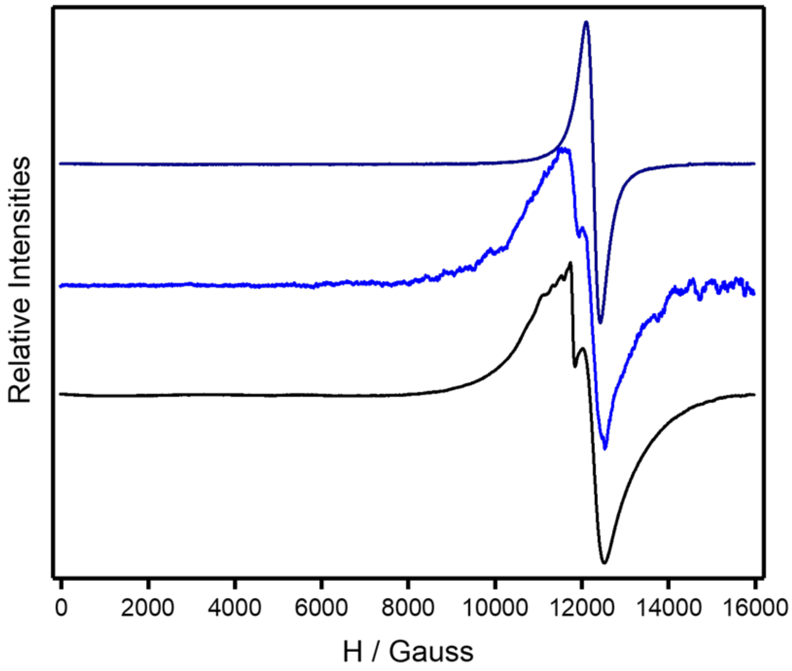

Figure S7. Continuous Wave Q-Band (ca. 34 GHz) EPR spectrum of **2** as a powder (black) 5K, solution (light blue) 5K and solution (navy blue) 50K.

Supplementary References

1. Rigaku Oxford Diffraction, CrysAlisPro Software system, version 40, Rigaku Corporation, Oxford, UK, **2017**.

2. G. M. Sheldrick, *Acta Cryst.* A, **2015**, *71*, 3-8.

3. O.V. Dolomanov, L. J. Bourhis, R. J. Gildea, J. A. L. Howard, H. Puschmann *J. Appl. Cryst.* **2009**, *42*, 339-341.

4. B. Bauer, L. D. Carr, H. G. Evertz, A. Feiguin, J. Freire, S. Fuchs, L. Gamper, J. Gukelberger, E. Gull, S. Guertler, A. Hehn, R. Igarashi, S. V. Isakov, D. Koop, P. N. Ma, P. Mates, H. Matsuo, O. Parcollet, G. Pawłowski, J. D. Picon, L. Pollet, E. Santos, V. W. Scarola, U. Schollwöck, C. Silva, B Surer, S. Todo, S. Trebst, M. Troyer, M. L. Wall, P. Werner, S. Wessel, *J. Stat. Mech*. 2011, P05001.

5. A. F. Albuquerque, F. Alet, P. Corboz, P. Dayal, A. Feiguin, S. Fuchs, L. Gamper, E. Gull, S. Gürtler, A. Honecker, R. Igarashi, M. Körner, A. Kozhevnikov, A. Läuchli, S. R. Manmana, M. Matsumoto, I. P. McCulloch, F. Michel, R. M. Noack, G. Pawlowski, L. Polett, T. Pruschke, U. Schollwöck, S. Todo, S. Trebst, M. Troyer, P. Werner, S. Wessel, *J. Mag. Mag. Mat.* 2007, **310**, 1187-1193.

6. S. Stoll, A. Schweiger, *J. Magn. Reson*. 2006, **178**, 42-55.
